# Giant “heart appearance-like sign” on MRI in bilateral ponto-medullary junction infraction: case report

**DOI:** 10.1186/s12883-020-01683-7

**Published:** 2020-03-23

**Authors:** Zhi-Hua Zhou, Yun-Fan Wu, Wei-Feng Wu, Ai-Qun Liu, Qing-Yun Yu, Zhong-Xing Peng, Ming-Fan Hong

**Affiliations:** 1 Department of neurology, The first affiliated hospital, School of Clinical Medicine of Guangdong Pharmaceutical University, Guangzhou, Guangdong China; 2 Department of Medical Imaging, Guangdong Second Provincial General Hospital, Guangzhou, China

**Keywords:** Medial medullary infarction (MMI), Medial pons infarction (MPI), Ponto-medullary junction infraction, Heart appearance, Magnetic resonance image (MRI), Digital subtraction angiography (DSA)

## Abstract

**Background:**

Bilateral medial medullary infarction (MMI) is uncommon and bilateral medial pons infarction (MPI) is even rarer. “Heart appearance” on magnetic resonance imaging (MRI) is a characteristic presentation of bilateral medial medullary infarction (MMI).

**Case presentation:**

We present 67-year-old Chinese diabetic and hypertensive female patient affected with “heart appearance-like” infarction in bilateral ponto-medullary junction on MRI. Abnormal signal was observed in the bilateral ponto-medullary junction on T1, T2, fluid-attenuated inversion recovery and apparent diffusion coefficient (ADC). The whole brain digital subtraction angiography (DSA) showed the basilar artery and vertebral artery remained intact. Therefore, we speculated that the bilateral ponto-medullary junction infarction might be caused by the deep perforating branch of the basilar artery.

**Conclusions:**

As far as we know, the “heart appearance-like” infraction in bilateral ponto-medullary junction was not reported. Our case also suggests that bilateral ischemic infraction involvement of the medulla and pon is possible even in the context of an intact basilar artery.

## Background

Bilateral medial medullary infarction (MMI) is uncommon, accounting for 0.5–1.51% of all strokes [1–3]. Bilateral medial pons infarction (MPI) is even rarer, accounting for <10% of all pontine infarctions [4]. “Heart appearance” on magnetic resonance imaging (MRI) is a characteristic presentation of bilateral medial medullary infarction (MMI) [5–7]. “Heart appearance” of the bilateral medial pons infarction (MPI) has been described rarely [4, 8, 9].

As far as we know, “heart appearance-like” infarction of the bilateral ponto-medullary junction has not been reported. In this paper, we present a patient affected with “heart appearance-like” infarction of the bilateral ponto-medullary junction on MRI and the whole brain digital subtraction angiography (DSA) showed the basilar artery (BA) and bilateral vertebral artery (VA) remained intact.

## Case presentation

A 67-year-old Chinese diabetic and hypertensive female patient presented with sudden onset vertigo with nausea and vomiting 4 days ago. Then she presented with sudden onset of right hemiparesis 2 days ago and developed rapidly quadriplegia (upper and lower limbs with grade 0 power), dysarthria, bilateral facial weakness. Her eyes moved normally and no diplopia. She had no history of smoking or drinking. On physical examination, the temperature was 36.8 °C, the pulse 76 beats per minute, and the blood pressure 165/95 mmHg. Detailed neurological examinations revealed quadriplegia, dysarthria, bilateral facial weakness, bilateral positive Babinski’s sign. The pupils were equal and reactive. Corneal reflexes were present and there was no gaze palsy and nystagmus, scoring 24 on the National Institute of Health Stroke Scale (NIHSS). On admission, laboratory findings indicated she had abnormal fasting blood sugar, HbA1c, total cholesterol (TC), triglyceride (TG), low-density lipoprotein cholesterol (LDL-c), uric acid. Detailed results were shown in Table 1. Twenty four hours Holter electrocardiography showed sinus rhythm without ST-T segment change and occasional premature atrial contractions (22 times within 24 h). Echocardiography showed mild mitral valve regurgitation and the left ventricular ejection fraction was 68%. Color Doppler flow imaging examination showed a mild resistance index of the intracranial segment of the vertebral artery (VA) and BA.

**Table 1:** Laboratory findings in the patient

Laboratory tests	Results	Normal range
Fasting blood sugar	12.6 mmol/L	3.89–6.1 mmol/L
2 h postprandial blood sugar	16.9 mmol/L	<7.8 mmol/L
HbA1c	12.5%	4–6%
Total cholesterol (TC)	6.55 mmol/L	3.5~5.69 mmol/L
Triglyceride (TG)	2.36 mmol/L	0.45~1.70 mmol/L
Low density lipoprotein cholesterol (LDL-c)	3.82 mmol/L	<3.12 mmol/L
High density lipoprotein cholesterol (HDL-c)	0.74 mmol/L	0.7~2.0 mmol/L
Uric acid	625 umol/L	90~360umol/L
Homocysteine	7.36	5~15 μmol/L
Prothrombin time (PT)	13.5 s	11.0~15.0 s
Activated partial thromboplastin time (APTT)	32.7 s	28.0~45.0 s
Thrombin time (TT)	17.8 s	14.0~21.0 s
International normalized ratio (INR)	1.01	0.8~1.2
Protein C	96%	60~140%
Protein S	98%	63.5~149%

**Fig. 1:**
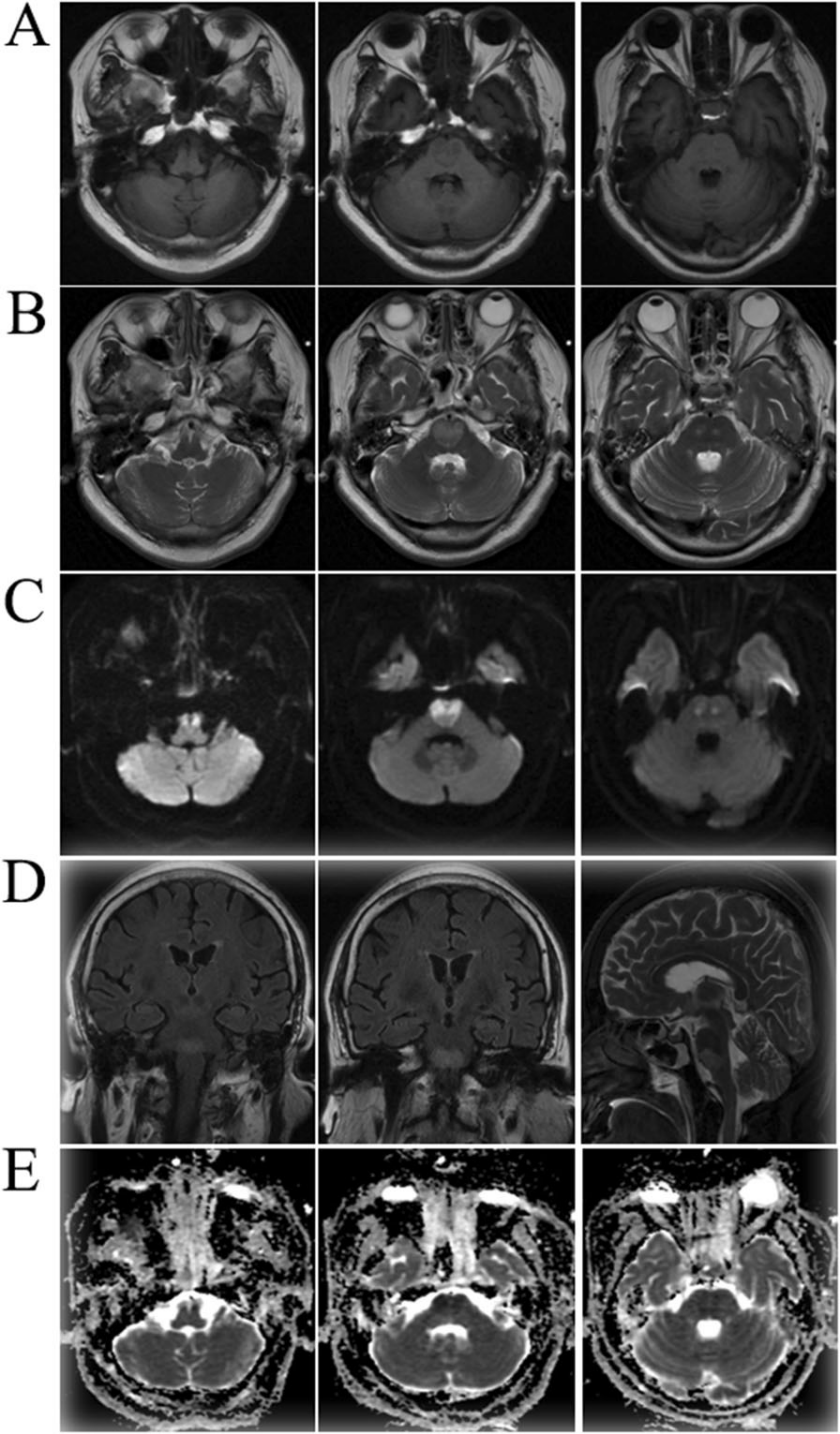
Cranial magnetic resonance imaging (MRI) indicated the giant “heart appearance” on the both sides in the ventral ponto-medullary junction. **a**, axial T1-MRI; **b**, axial T2-MRI; **c**, axial diffusion-weighted image MRI (DWI); **d**, coronal fluid attenuated inversion recovery (FLAIR) and Sagittal T2-MRI; **e**, axial apparent diffusion coefficient map MRI (ADC). The “heart appearance” lesion shows hypointensity on T1 and ADC, hyperintensity on T2, DWI and FLAIR, indicating, in conjunction with the finding on DWI, that the lesion is a subacute infarct

**Fig. 2:**
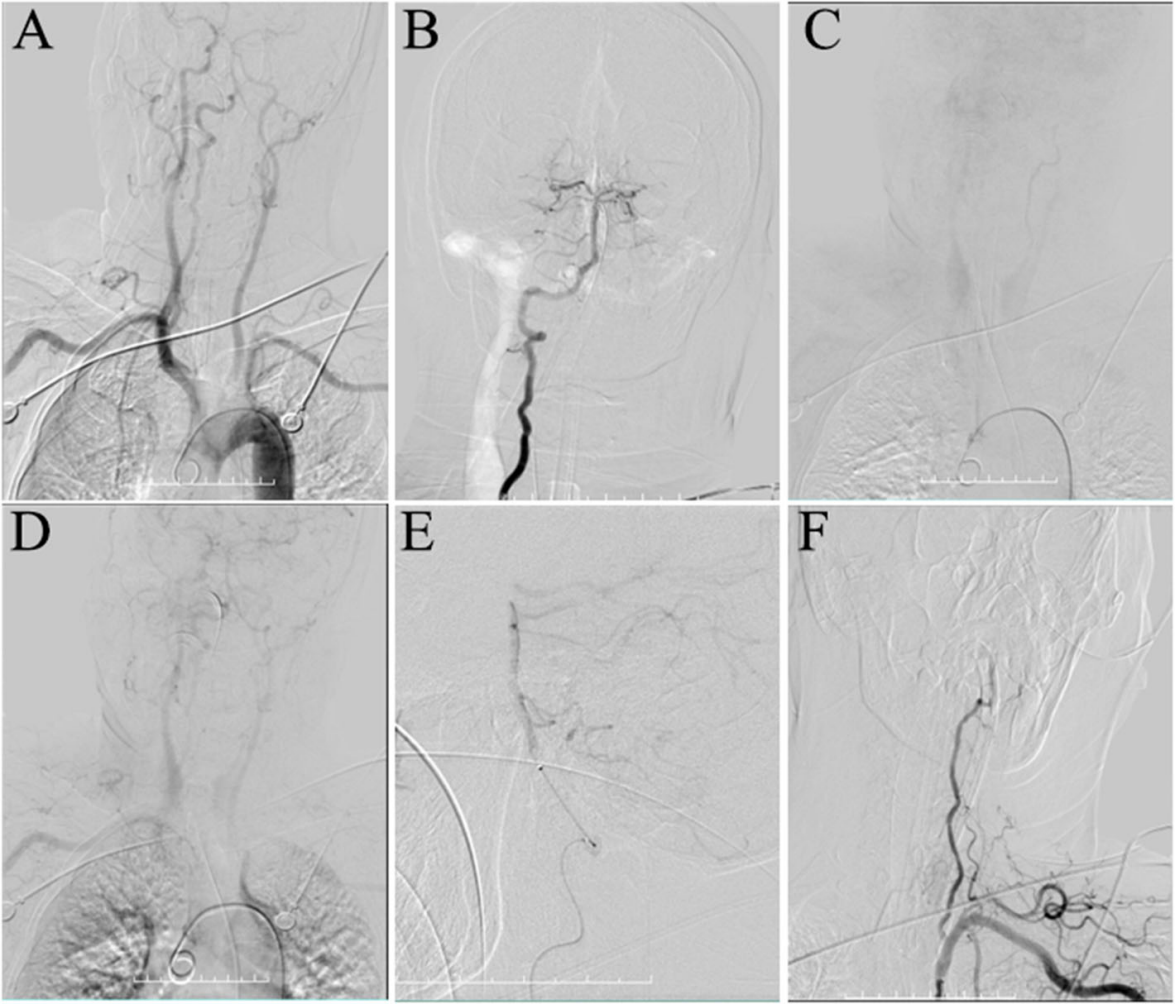
Digital subtraction angiography (DSA) indicated the vertebral artery and the basilar artery remains intact despite the bilateral involvement of the ponto-medullary junction. The basilar artery and the right vertebral artery are intact (**a**, **b**). The left vertebral artery is slender (**a**, **c**, **d**, **e**, **f**)

Cranial magnetic resonance imaging (MRI) brain diffusion-weighted imaging at 3.0 T revealed a giant heart-shaped hyperintensity area (“heart appearance-like sign”) on both sides in the ventral ponto-medullary junction [Fig. 1]. Abnormal signal was observed in the same region by T1, T2, fluid-attenuated inversion recovery and apparent diffusion coefficient (ADC). On the basis of these findings, the patient was diagnosed to be having an acute bilateral ponto-medullary junction infarction. Next, we executed the detection of whole brain digital subtraction angiography (DSA) for the patient and DSA showed the basilar artery and vertebral artery remained intact [Fig. 2]. Therefore, we speculated that the bilateral ponto-medullary junction infarction might be caused by the deep perforating branch of the basilar artery. The patient was treated with Aspirin (100 mg/d) and rehabilitation was initiated. Six months later, the patient still had quadriplegia (upper and lower limbs with grade 2 power), dysarthria, bilateral facial weakness and nasal feeding.

## Discussion and conclusions

Bilateral MMI or bilateralis MPI is a rare stroke syndrome [4, 10]. Recently, the “heart appearance” infraction in bilateral medial medullary and bilateral pons has been described gradually [5, 8, 9]. Here we present a 67-year-old Chinese diabetic and hypertensive female patient affected with “heart appearance-like” infarction of the bilateral ponto-medullary junction on MRI findings. To the best of our knowledge, giant “heart appearance-like” infarction of the bilateral ponto-medullary junction has not been reported.

MRI findings indicated “heart appearance-like” acute ischemic infarction of the bilateral ponto-medullary junction. DSA findings indicated the VA and BA remained intact. So we speculated that the bilateral ponto-medullary junction infarction might be caused by the deep perforating branch, short circumflex branch of basilar artery of the basilar artery. Combined with the history of the patient, which she had a history of diabetes and hypertension and laboratory tests revealed abnormal lipid metabolism and hyperuricemia on admission, we postulated microatheromatosis affecting the perforator branches of the basilar artery, including basilar artery branch disease, or small artery disease as the underlying mechanism. Arterial supply of brainstem is divided into the anteromedial territory, anterolateral territory, lateral territory, and posterior territory [11]. The “heart appearance-like” sign is considered to appear when the infarct occurs in the anteromedial and anterolateral territories. Since the scheme of vascularization of the pons is identical to that of the medulla [4], we also hold that it should not be surprising to encounter “heart appearance” infarction of the pons [9]. The overall outcome of this type of stroke is poor [10], so we need to early recognition and treatment. Our patient ended up with quadriplegia (upper and lower limbs with grade 2 power), dysarthria, bilateral facial weakness and nasal feeding.

As far as we know, the “heart appearance-like” infraction in bilateral ponto-medullary junction infarction was not reported. Our case also suggests that bilateral ischemic infraction involvement of the medulla and pon is possible even in the context of an intact basilar artery [9].

## Data Availability

The datasets used and/or analysed during the current study are available from the corresponding author on reasonable request.

## Abbreviations

MMI: Medial medullary infarction
MPI: Medial pons infarction
MRI: Magnetic resonance image
DSA: Digital subtraction angiography
